# Draft Metagenomes of Endolithic Cyanobacteria and Cohabitants from Hyper-Arid Deserts

**DOI:** 10.1128/MRA.00206-21

**Published:** 2021-07-29

**Authors:** Bayleigh Murray, Micah Dailey, Emine Ertekin, Jocelyne DiRuggiero

**Affiliations:** a Johns Hopkins University, Department of Biology, Baltimore, Maryland, USA; b Johns Hopkins University, Department of Earth and Planetary Sciences, Baltimore, Maryland, USA; Montana State University

## Abstract

Cyanobacteria are essential to microbial communities inhabiting translucent rocks in hyper-arid deserts. Metagenomic studies revealed unique adaptations of these cyanobacteria, but validation of the corresponding metabolic pathways remained challenging without access to isolates. Here, we present high-quality metagenome-assembled genomes for cyanobacteria, and their heterotrophic companions, isolated from endolithic substrates.

## ANNOUNCEMENT

In the most arid deserts, where environmental conditions are extreme, microbial communities find refuge inside rocks as a survival strategy (1). The rock habitat protects microorganisms from high UV radiation and drastic temperature fluctuations and promotes water retention within the rock matrix (2). Molecular studies of endolithic communities (within rock) revealed ecosystems spanning all domains of life and multiple trophic levels (3–5). The communities are based on the primary production of cyanobacteria, and sometimes algae, and are constituted of an assemblage of heterotrophic bacteria and/or archaea and viruses (6–10). Endolithic communities are highly specific to their lithic substrate, with fine-scale diversification of the microbial reservoir driven by substrate properties (3, 10).

Cyanobacteria inhabiting endolithic substrates in arid deserts are mostly members of the orders *Chroococcales* (*Chroococcidiopsis* and *Gloeocapsa*), *Nostocales*, and *Oscillatoriales* (1). Metagenomic studies of endolithic communities revealed unique adaptations of these cyanobacteria, and a large number of pathways for secondary metabolites, nonribosomal peptides, and polyketides are encoded in their genomes (7, 10). However, validation of these pathways remained challenging without access to isolates. Here, we present the metagenome-assembled genomes (MAGs) of cyanobacteria isolated from endolithic substrates collected in the Atacama and Negev Deserts (Table 1). Because these isolates are not purified cultures, their companions—heterotrophic bacteria—were also sequenced.

**TABLE 1:** Metagenome and MAG statistics of endolithic cyanobacterial isolates from the Atacama Desert, Chile, and the Negev Desert, Israel

Sample name	Substrate	IMG taxon ID	Metagenome size (Mbp)	Bin ID	Taxon/genus	MAG completion (%)	MAG contamination (%)	MAG size (Mbp)	MAG gene count	MAG scaffold count
C-VL-3P3	Calcite	3300039404	11	3300039404_1	*Chroococcidiopsis*	99.48	1.63	6.6	6,630	157
				3300039404_2	*Deinococcus*	97.67	0.99	4.1	4,214	68
G-Km37-3P1	Gypsum	3300039405	18.2	3300039405_1	*Methylobacterium*	100	0	6.9	6,942	64
				3300039405_2	*Deinococcus*	97.67	0.99	4.1	4,212	67
G-Km37-3P3	Gypsum	3300039416	42.9	3300039416_1	*Chroococcidiopsis*	99.48	1.63	6.6	6,618	153
				3300039416_2	*Deinococcus*	97.67	0.99	4.1	4,213	66
G-MTQ-3P1	Gypsum	3300038622	16.2	3300038622_1	*Chroococcidiopsis*	99.48	1.63	6.6	6,601	163
				3300038622_2	*Methylobacterium*	52.45	1.25	4.2	4,745	668
G-MTQ-3P2	Gypsum	3300037877	9.9	3300037877_1	*Chroococcidiopsis*	99.48	1.63	6.6	6,608	161
H-SG-1P1	Gypsum	3300039034	38.8	3300039034_1	*Chroococcidiopsis*	99.48	1.63	6.6	6,605	160
H-SG-2P1	Gypsum	3300039035	43.1	3300039035_2	*Chroococcidiopsis*	99.48	1.63	6.6	6,619	155
				3300039035_3	*Deinococcus*	97.67	0.99	4.1	4,230	70
I-MTQ-2P3	Ignimbrite	3300039417	20	3300039417_1	*Chroococcidiopsis*	97.11	4.52	7.6	7,825	531
				3300039417_2	*Deinococcus*	98.52	0.99	4.2	4,428	95
				3300039417_3	*Thermomicrobiales*	63.91	1.89	2.4	2,800	503
I-MTQ-3P1	Ignimbrite	3300039418	28.2	3300039418_3	*Deinococcus*	97.67	0.99	4.1	4,241	70
I-MTQ-3P3	Ignimbrite	3300039424	30.6	3300039424_2	*Aquamicrobium*	99.59	0.75	4.4	4,417	7
				3300039424_3	*Deinococcus*	97.25	0.99	4.1	4,315	99
				3300039424_4	*Microcella*	99.38	0.25	2.5	2,464	5
I-MTQ-4P3	Ignimbrite	3300039425	10.3	3300039425_1	*Deinococcus*	97.67	0.99	4.1	4,237	70
S-NGV-2P1	Sandstone	3300039401	43	3300039401_1	*Chroococcidiopsis*	99.48	1.63	6.6	6,617	153
				3300039401_2	*Deinococcus*	97.67	0.99	4.1	4,214	67
S-NGV-2P2	Sandstone	3300039032	6.8	3300039032_1	*Chroococcidiopsis*	99.48	1.63	6.5	6,596	163
S-NGV-3P2	Sandstone	3300039033	6.8	3300039033_1	*Chroococcidiopsis*	99.48	1.63	6.6	6,618	158

Cyanobacterial isolates were obtained by incubating ground colonized rock samples collected in the Atacama and Negev Deserts (3, 4) in Bold’s basal medium (11) and in BG11 liquid medium (12) for 5 weeks at 25°C under 24 μM photons/m^2^/s of white light (WL) using Philips daylight deluxe linear fluorescent T12 40-W light bulbs and a combination of neutral-density filters (299 1.2ND and 298 0.15ND; Lee Filters, Burbank, CA). Single colonies from 1% agar BG11 plates were then transferred to liquid BG11 medium and grown under WL; it is important to note that these were not anoxic cyanobacterial cultures but, rather, a mixture of cyanobacteria and heterotrophic bacteria. Total DNA was extracted from cell pellets using the PowerSoil DNA extraction kit (MoBio Laboratories, Inc., Solana Beach, CA). Nextera libraries, with Ranger size technology, were made with total DNA and sequenced to a 2-Gb depth using 2 × 150-nucleotide (nt) reads on an Illumina NovaSeq instrument at the Department of Energy (DOE) Joint Genome Institute (JGI). Sequence quality control was performed with the BBTools package (https://jgi.doe.gov/data-and-tools/bbtools/), and sequence reads were assembled with metaSPAdes version 3.13.0 using the “metagenome” flag and running the assembly module without error correction and with kmer sizes 33, 55, 77, 99, and 127 (13). MetaBAT v2.12.1 (14) was used for binning. MAGs were evaluated with CheckM v1.0.12 (15) and annotated with GTDB-Tk version v0.2.2 and the GTDB database release 86 (16). Default parameters were used for all software unless otherwise noted. Only high-quality (HQ) and medium-quality (MQ) bins were reported based on Minimum Information about a Metagenome-Assembled Genome (MIMAG) standards (17).

High-quality MAGs of cyanobacteria, together with MAGs of heterotrophic bacteria, were recovered from most samples (Table 1). All cyanobacteria belonged to the *Chroococcidiopsis* genus; *Deinococcus* was the most common heterotrophic bacterium, but we also found members of the *Proteobacteria*, *Actinobacteria*, and *Chloroflexi*, illustrating the diversity of these communities.

### Data availability.

The raw sequencing data are available from the National Centre for Biotechnology Information under BioProject numbers PRJNA654119, PRJNA654120, PRJNA654121, PRJNA654122, PRJNA654123, PRJNA654124, PRJNA677471, PRJNA677472, PRJNA677473, PRJNA677474, PRJNA677475, PRJNA677476, PRJNA677477, and PRJNA677478. The metagenome coassembly and functional annotation are available from the JGI Genome Portal under the IMG taxon IDs reported in Table 1. To obtain cultures of cyanobacterial isolates, please contact the corresponding author.

## References

[1] Meslier V, DiRuggiero J. 2019. Endolithic microbial communities as model systems for ecology and astrobiology, p 145–168. *In* Seckbach J, Rampelotto P (ed), Model ecosystems in extreme environments. Academic Press, San Diego, CA. doi:10.1016/B978-0-12-812742-1.00007-6.

[2] Walker JJ, Pace NR. 2007. Endolithic microbial ecosystems. Annu Rev Microbiol 61:331–347. doi:10.1146/annurev.micro.61.080706.093302. 17506683

[3] Meslier V, Casero MC, Dailey M, Wierzchos J, Ascaso C, Artieda O, McCullough PR, DiRuggiero J. 2018. Fundamental drivers for endolithic microbial community assemblies in the hyperarid Atacama Desert. Environ Microbiol 20:1765–1781. doi:10.1111/1462-2920.14106. 29573365

[4] Qu EB, Omelon CR, Oren A, Meslier V, Cowan DA, Maggs-Kolling G, DiRuggiero J. 2019. Trophic selective pressures organize the composition of endolithic microbial communities from global deserts. Front Microbiol 10:2952. doi:10.3389/fmicb.2019.02952. 31969867 PMC6960110

[5] Wierzchos J, DiRuggiero J, Vítek P, Artieda O, Souza-Egipsy V, Skaloud P, Tisza M, Davila AF, Vílchez C, Garbayo I, Ascaso C. 2015. Adaptation strategies of endolithic chlorophototrophs to survive the hyperarid and extreme solar radiation environment of the Atacama Desert. Front Microbiol 6:934. doi:10.3389/fmicb.2015.00934. 26441871 PMC4564735

[6] Crits-Christoph A, Gelsinger DR, Ma B, Wierzchos J, Ravel J, Ascaso C, Artieda O, Davila A, DiRuggiero J. 2016. Functional interactions of archaea, bacteria and viruses in a hypersaline endolithic community. Environ Microbiol 18:2064–2077. doi:10.1111/1462-2920.13259. 26914534

[7] Crits-Christoph A, Robinson CK, Ma B, Ravel J, Wierzchos J, Ascaso C, Artieda O, DiRuggiero J. 2016. Phylogenetic and functional substrate specificity for endolithic microbial communities in hyper-arid environments. Frontiers Microbiol 7:301. doi:10.3389/fmicb.2016.00301. PMC4784552 27014224

[8] Uritskiy G, Getsin S, Munn A, Gomez-Silva B, Davila A, Glass B, Taylor J, DiRuggiero J. 2019. Halophilic microbial community compositional shift after a rare rainfall in the Atacama Desert. ISME J 13:2737–2749. doi:10.1038/s41396-019-0468-y. 31273300 PMC6794293

[9] Uritskiy G, Tisza MJ, Gelsinger DR, Munn A, Taylor J, DiRuggiero J. 2020. Cellular life from the three domains and viruses are transcriptionally active in a hypersaline desert community. Environ Microbiol doi:10.1111/1462-2920.15023. 32307861

[10] Ertekin E, Meslier V, Browning A, Treadgold J, DiRuggiero J. 2021. Rock structure drives the taxonomic and functional diversity of endolithic microbial communities in extreme environments. Environ Microbiol doi:10.1111/1462-2920.15287. 33078515

[11] Cox ER, Bold HC. 1966. Taxonomic investigation of Stigeoclonium. *In* Phycological studies VII, vol 10. University of Texas, Austin, Texas.

[12] Rippka R, Deruelles J, Waterbury JB, Herdman M, Stainer RY. 1979. Generic assignments, strain histories and properties of pure cultures of cyanobacteria. J Gen Microbiol 111:1–61. doi:10.1099/00221287-111-1-1.

[13] Bankevich A, Nurk S, Antipov D, Gurevich AA, Dvorkin M, Kulikov AS, Lesin VM, Nikolenko SI, Pham S, Prjibelski AD, Pyshkin AV, Sirotkin AV, Vyahhi N, Tesler G, Alekseyev MA, Pevzner P. 2012. SPAdes: a new genome assembly algorithm and its applications to single-cell sequencing. J Comput Biol 19:455–477. doi:10.1089/cmb.2012.0021. 22506599 PMC3342519

[14] Kang DD, Froula J, Egan R, Wang Z. 2015. MetaBAT, an efficient tool for accurately reconstructing single genomes from complex microbial communities. PeerJ 3:e1165. doi:10.7717/peerj.1165. 26336640 PMC4556158

[15] Parks DH, Imelfort M, Skennerton CT, Hugenholtz P, Tyson GW. 2015. CheckM: assessing the quality of microbial genomes recovered from isolates, single cells, and metagenomes. Genome Res 25:1043–1055. doi:10.1101/gr.186072.114. 25977477 PMC4484387

[16] Chaumeil P-A, Mussig AJ, Hugenholtz P, Parks DH. 2019. GTDB-Tk: a toolkit to classify genomes with the Genome Taxonomy Database. Bioinformatics 36:1925–1927. doi:10.1093/bioinformatics/btz848. PMC7703759 31730192

[17] Bowers RM, Kyrpides NC, Stepanauskas R, Harmon-Smith M, Doud D, Reddy TBK, Schulz F, Jarett J, Rivers AR, Eloe-Fadrosh EA, Tringe SG, Ivanova NN, Copeland A, Clum A, Becraft ED, Malmstrom RR, Birren B, Podar M, Bork P, Weinstock GM, Garrity GM, Dodsworth JA, Yooseph S, Sutton G, Glöckner FO, Gilbert JA, Nelson WC, Hallam SJ, Jungbluth SP, Ettema TJG, Tighe S, Konstantinidis KT, Liu W-T, Baker BJ, Rattei T, Eisen JA, Hedlund B, McMahon KD, Fierer N, Knight R, Finn R, Cochrane G, Karsch-Mizrachi I, Tyson GW, Rinke C, Lapidus A, Meyer F, Yilmaz P, Parks DH, Eren AM, Genome Standards Consortium, et al. 2017. Minimum information about a single amplified genome (MISAG) and a metagenome-assembled genome (MIMAG) of bacteria and archaea. Nat Biotechnol 35:725–731. doi:10.1038/nbt.3893. 28787424 PMC6436528

